# Double-donor complex in vertically coupled quantum dots in a threading magnetic field

**DOI:** 10.1186/1556-276X-7-531

**Published:** 2012-09-26

**Authors:** Ramón Manjarres-García, Gene Elizabeth Escorcia-Salas, Javier Manjarres-Torres, Ilia D Mikhailov, José Sierra-Ortega

**Affiliations:** 1 Group of Investigation in Condensed Matter Theory, Universidad del Magdalena, Santa Marta, Colombia; 2 Universidad Industrial de Santander, A. A. 678, Bucaramanga, Colombia

**Keywords:** Quantum dots, Adiabatic approximation, Artificial molecule, PACS, 78.67.-n, 78.67.Hc, 73.21.-b

## Abstract

We consider a model of hydrogen-like artificial molecule formed by two vertically coupled quantum dots in the shape of axially symmetrical thin layers with on-axis single donor impurity in each of them and with the magnetic field directed along the symmetry axis. We present numerical results for energies of some low-lying levels as functions of the magnetic field applied along the symmetry axis for different quantum dot heights, radii, and separations between them. The evolution of the Aharonov-Bohm oscillations of the energy levels with the increase of the separation between dots is analyzed.

## Background

An important feature in low-dimensional systems is the electron-electron interaction because it plays a crucial role in understanding the electrical transport properties of quantum dots (QDs) at low temperatures [1]. Such systems may involve small or large numbers of electrons as well as being confined in one or more dimensions. The number of electrons in a QD can be varied over a considerable range. It is possible to control the size and the number of electrons and to observe their spatial distributions in QDs. Energy spectrum of two-electron QD with a parabolic confinement, for which two-particle wave equation can be separated completely, has been analyzed previously by using different methods [2-5].

In the present work, we propose another exactly solvable two-electron heterostructure in which two separated electrons are confined in vertically coupled QDs with a special lens-like morphology. Together with two on-axis donors, these two electrons generate an artificial hydrogen-like molecule whose properties can be controlled by varying the geometric parameters and the strength of the magnetic field applied along the symmetry axis.

## Methods

**Figure 1:**
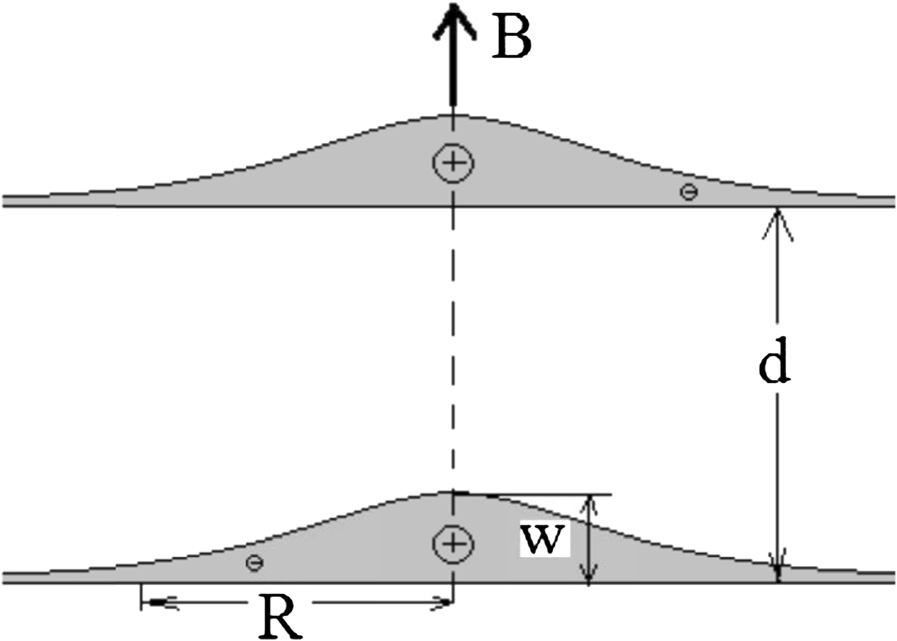
Scheme of the artificial hydrogen-like molecule.

The model which we analyze below consists of two identical, axially symmetrical and vertically coupled QDs with the on-axis donor located in each one of them (see Figure 1). The dimension of the heterostructure is defined by the QDs' radii *R,* height *W*, and the separation *d* between them along the *z*-axis. We assume that the QDs have a shape of very thin layers whose profiles are given by the following dependence of the thickness of the layers *w* on the distance *ρ* from the axis:

(1) $$w\left(\rho \right)=W/\sqrt{1+{\left(\rho /R\right)}^{2}}$$

Besides, for the sake of simplicity, we consider a model with infinite barrier confinement, which is defined in cylindrical coordinates as $V\left(r\right)=0$ if $0<z<w\left(\rho \right)$, and $V\left(r\right)=\infty$ otherwise.

Given that the thicknesses of the layers are much smaller than their lateral dimensions, one can take advantage of the adiabatic approximation in order to exclude from consideration the rapid particle motions along the *z*-axis [6,7] and obtain the following expression for effective Hamiltonian in polar coordinates:

(2) $$H=\sum_{i=1,2}H_{0}\left(\rho_{i}\right)+V\left(\rho_{1},\rho_{2}\right)+2\frac{\pi^{2}}{W^{2}};H_{0}\left(\rho_{i}\right)=-\Delta_{i}^{\left(2D\right)}+i\gamma \frac{\partial }{\partial \vartheta_{i}}+\frac{\omega^{2}\rho_{i}^{2}}{4};\omega^{2}={\left(2\pi /W\cdot R\right)}^{2}+\gamma^{2}V\left(\rho_{1},\rho_{2}\right)=\frac{2}{\sqrt{d{\;}^{2}+{\left|\rho_{1}-\rho_{2}\right|}^{2}}}-\sum_{i=1,2}\left[\frac{2}{\sqrt{d{\;}^{2}+{\left|\rho_{i}\right|}^{2}}}+\frac{2}{\left|\rho_{i}\right|}\right]$$

The effective Bohr radius $a_{0}^{*}=\hbar^{2}\epsilon /m*e^{2}$ as the unit of length, the effective Rydberg $Ry*=e^{2}/2\epsilon \;a_{0}^{*}=\hbar^{2}/2m*{a_{0}^{*}}^{2}$ as the energy unit, and γ=eℏB/2m*cRy* as the unit of the magnetic field strength have been used in Hamiltonian (Equation 2), with m* being the electron effective mass and *ϵ*, the dielectric constant. The polar coordinates $\rho_{k}=\left(\rho_{k},\vartheta_{k}\right)$ labeled by k=1,2 correspond to the first and the second electrons, respectively. It is seen that for the selected particular profile given by Equation 1, the Hamiltonian (Equation 2) coincides with one which describes two particles in 2D quantum dot with parabolic confinement and renormalized interaction. It is well known that such Hamiltonian may be separated by using the center of mass, $R=\left(\rho_{1}+\rho_{2}\right)/2$, and the relative, $\rho =\rho_{1}-\rho_{2}$ coordinates [8]:

(3) $$\begin{array}{l} H=H_{R}+2H_{\rho };\;H_{R}=-\frac{\Delta_{R}^{\left(2D\right)}}{2}+\frac{1}{2}\omega^{2}R^{2};\\ H_{\rho }=-\Delta_{\rho }^{\left(2D\right)}+\frac{\omega^{2}\rho^{2}}{16}-\frac{3}{\rho }-\frac{4}{\sqrt{\rho^{2}+4d^{2}}} \end{array}$$

The wave function is factorized into two parts, $\psi \left(R,\rho \right)=\Phi \left(R\right)\varphi \left(\rho \right)$, describing the center of mass and the relative motions, respectively. Meanwhile, the total energy splits into two terms depending on two radial $N_{R},n_{\rho }$ and two azimuthal $L_{R},l_{\rho }$ quantum numbers:

(4) $$E\left(N_{R},L_{R};n_{\rho },l_{\rho }\right)=E_{R}\left(N_{R},L_{R}\right)+2E_{\rho }\left(n_{\rho },l_{\rho }\right)=\left(2N_{R}+L_{R}\right)\omega +2E_{\rho }\left(n_{\rho },l_{\rho }\right)$$

where the first term represents the well-known expression for the exact energy levels of a two-dimensional harmonic oscillator, labeled by the radial $\left(N_{R}=0,\;1,\;2,\;\ldots \right)$ and azimuthal $\left(L_{R}=0\;\pm \;1\;\pm \;2,\;\ldots \right)$ quantum numbers for the center of mass motion and the relative motion energy $2E_{\rho }\left(n_{\rho },l_{\rho }\right)$ must be found solving the following one-dimensional Schrödinger equation:

(5) $$-u''(\rho )+V(\rho )u(\rho )=E_{\rho }(n_{\rho },l_{\rho })u(\rho );V(\rho )=\omega^{2}\rho^{2}/4+\left(l_{\rho }^{2}-1/4\right)/\rho^{2}-3/\rho -4/\sqrt{\rho^{2}+4d^{2}}$$

In our numerical, work the trigonometric sweep method [8] is used to solve this equation.

## Results and discussion

Before the results are shown and discussed, it is useful to specify the labeling of quantum levels of the two-electron molecular complex. According to Equation 4, the energy levels $E\left(N_{R},L_{R};n_{\rho },l_{\rho }\right)$ can be labeled by four symbols $N_{R},L_{R};n_{\rho },l_{\rho }$*.* Even and odd *l*_*p*_ correspond to the spin singlet and triplet states, respectively, consistent with the Pauli Exclusion Principle.

**Figure 2:**
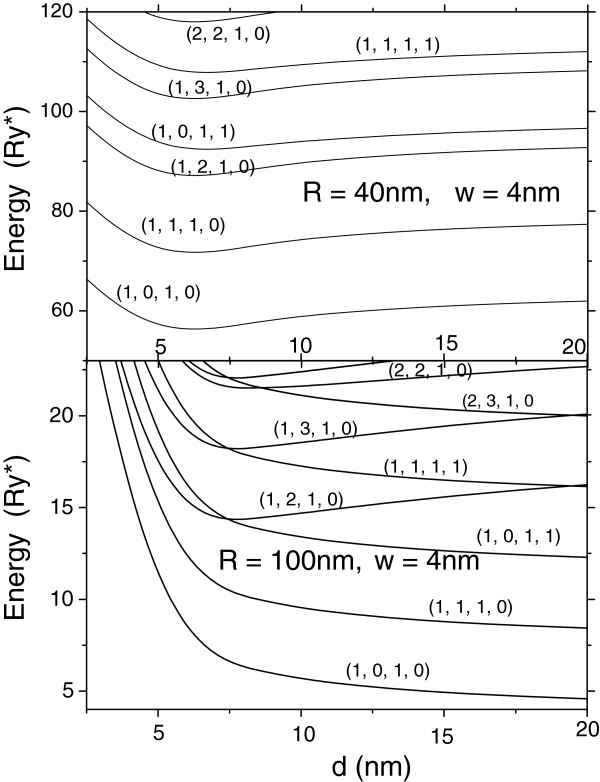
**Energies**$\tilde{E}\left(d\right)$**of the double-donor complex corresponding to some low-lying levels in vertically coupled QDs.** As functions of the distance between them.

We have performed numerical calculations of energy levels of complexes with radii *R* between 20 and 100 nm for different separations between layers. In all presented calculation results, the top thickness *W* is taken as 0.4 nm. In order to highlight the role of the interplay between the quantum size and correlation effects in the formation of the energy spectrum of our artificial system different from the natural hydrogen molecular complex, we have plotted in Figure 2 the potential curves $\tilde{E}\left(d\right)=E\left(N_{R},L_{R};n_{\rho },l_{\rho }\right)+2/d$, similar to those of the hydrogen molecule in which the complex energies with the electrostatic repulsion between donors included as functions of the separation *d* between QDs are shown. Comparing them with the corresponding potential curves of the hydrogen molecule, one can to take into account that in analyzing the structure here, the electron motion in contrast to hydrogen molecule is restricted inside two separated thin layers. The energy dependencies of different levels **(**labeled by four quantum numbers, $\left(N_{R},L_{R};n_{\rho },l_{\rho }\right)$ are shown in Figure 2 for QDs with two different radii, *R* = 40 nm and *R* = 100 nm. A clear difference in the behavior of the potential curves is readily seen. If the curves are smooth without any crossovers for QDs of small radius, the corresponding potential curves suffer a drastic change as the QD radius becomes large. In the last case, the energy levels become very sensitive to the variation of the separation between QDs, and the quantum size effect becomes essential, providing alteration of the energy gaps, multiple crossovers of levels with the same or different spins, and the level reordering, as the distance between QDs increases from 5 to 20 nm.

We ascribe a dramatic alteration of the potential curves with the increase of the separation between QDs from 5 to 20 nm observed in Figure 2 to the interplay between the structural confinement and the electron-electron repulsion. As the QDs' radii are small(*R* → 0), the confinement is strong, and the kinetic energy (~1/*R*^2^) is larger than the electron-electron repulsion energy (~1/*R*), vice versa for QDs with large radii. Therefore, as the QDs' radii increase, the arrangement of the electronic structure for different energy levels changes from typical for the gas-like system to crystal-like one, accompanied by the crossovers of the curves and reordering of the levels. As the two-electron structure arrangement for large separation between electrons becomes almost rigid, the relative motion of electrons is frozen out, and the two-electron structure transforms into a rigid rotator with practically fixed separation between electrons. The electrons' motion in this case becomes similar to one in 1D ring, and therefore, the energy dependencies on the external magnetic field applied along the symmetry axis should be similar to those which exhibit the Aharonov-Bohm effect.

**Figure 3:**
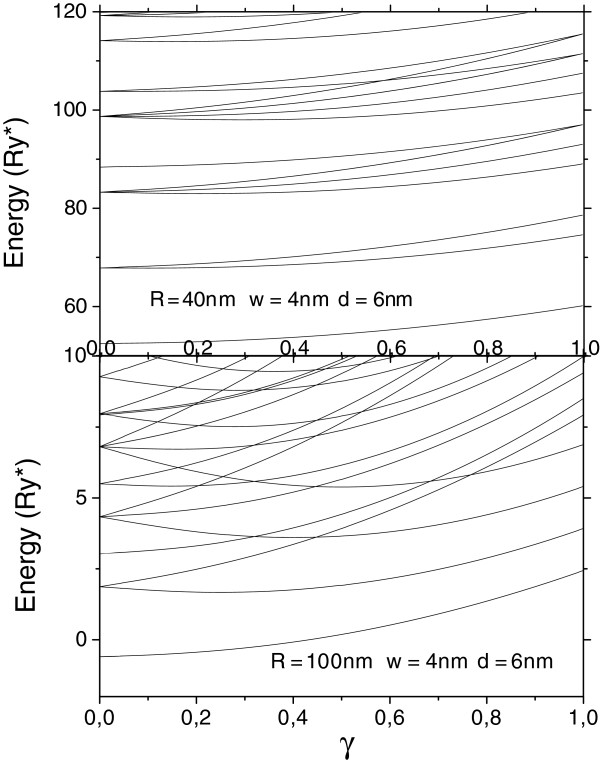
**Energies**$E\left(N_{R},L_{R};n_{\rho },l_{\rho }\right)$**of some low-lying levels of the double-donor complex in vertically coupled QDs.** As functions of the magnetic field.

In order to verify this hypothesis, we present in Figure 3 the calculated molecular complex energies $E\left(N_{R},L_{R};n_{\rho },l_{\rho }\right)$ of some lower levels as functions of the magnetic field strength for QDs with small *R* = 40 nm (upper curves) and large *R* = 100 nm radii (lower curves).

It is seen that for QD of small radius, the energies are increased smoothly with very few intersections. Such dependence is typical for gas-like systems where the paramagnetic term contribution is depreciable in comparison with the diamagnetic one. On the contrary, the energy dependency curves for QD of large radius present multiple crossovers and level-ordering inversion as the magnetic field strength increases from 0 to 1. It is due to a competition between diamagnetic (positive) and paramagnetic (negative) terms of the Hamiltonian whose contributions in total two-electron energy in QDs of large radii are of the same order while the electron arrangement is similar to a rigid rotator. In other words, the correlation in this case becomes as strong as the electrons are mainly located on the opposite sides within a narrow ring-like region.

**Figure 4:**
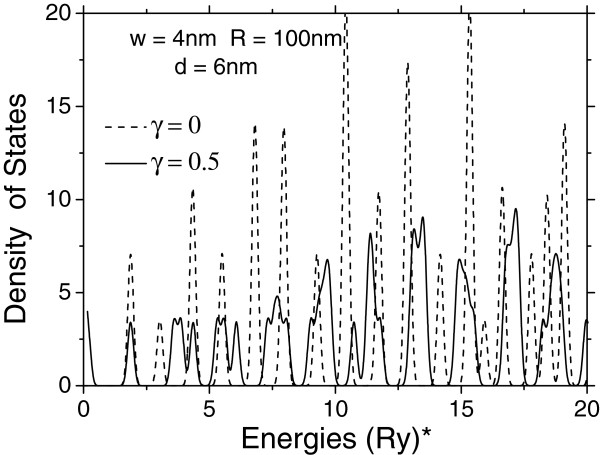
**Density of states for two different values of the magnetic field.** Corresponding to low-lying levels of the double-donor complex in vertically coupled QDs.

**Figure 5:**
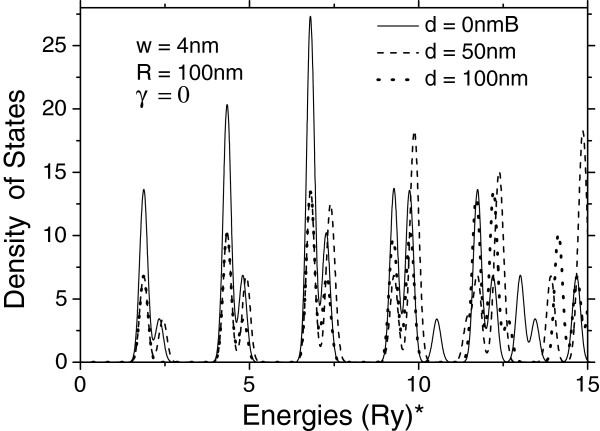
**Density of states for three different distances between layers.** Corresponding to low-lying levels of the double-donor complex in vertically coupled QDs.

Finally, in Figures 4 and 5, we present results of the calculation of the density of electronic states for double-donor molecular complex confined in vertically coupled QDs. It is clear from the discussion above that the presence of the magnetic field should provide a significant change of the density of the electronic states as the QDs' radii are sufficiently large. Indeed, it is seen from Figure 4 that under relatively weak magnetic field (γ = 0.5), as the molecular complex is confined in QDs of 100-nm with 6-nm separation between them, the density of states becomes essentially more homogeneous since the widths of individual lines are broadened and the gaps between them are reduced. Such change of the density of states is observed due to a splitting and displacement of the individual lines accompanied by their crossovers and the reordering of the energy levels.

In Figure 5, we present similar curves of the molecular complex density of states for three different separations between QDs. It is seen that the curves of the density of states are modified only slightly, essentially less than under variation of the magnetic field. Particularly, the lower energy peak positions are almost insensitive to any change of the distance between dots, while the upper energy peaks are noticeably displaced toward higher energy regions.

## Conclusions

In short, we propose a simple numerical procedure for calculating the energies and wave functions of a molecular complex formed by two separated on-axis donors located at vertically coupled quantum dots with a particular lens-type morphology which produces in-plane parabolic confinement. We show that in the adiabatic approximation, the Hamiltonian of this two-electron system included in the presence of the external magnetic field is separable. The curves of the energy dependencies on the external magnetic field and the separation between quantum dots are presented. Analyzing the curves of the low-lying energies as functions of the magnetic field applied along the symmetry axis, we find that the two-electron configuration evolves from one similar to a rigid rotator to gas-like as the dot radii decrease. This quantum size effect is accompanied by a significant modification of the density of the energy states and the energy dependencies on the external magnetic field and geometric parameters of the structure.

## Competing interests

The authors declare that they have no competing interests

## Authors' contributions

All authors contributed equally to this work. JSO created the analytic model with contributions from IM, RMG, and JMT. GES performed the numerical calculations and wrote the manuscript. All authors discussed the results and implications, commented on the manuscript at all stages, and read and approved the final manuscript.

## References

[B1] Kramer B Proceedings of a NATO Advanced Study Institute on Quantum Coherence in Mesoscopic System: 1990 April 2-13; Les Arcs, France 1991 New York: Plenum

[B2] Maksym PA Chakraborty T Quantum dots in a magnetic field: role of electron–electron interactions Phys Rev Lett 1990 65 108 111 10.1103/PhysRevLett.65.108 10042184

[B3] Pfannkuche D Gudmundsson V Maksym P Comparison of a Hartree, a Hartree-Fock, and an exact treatment of quantum-dot helium Phys Rev B 1993 47 2244 2250 10.1103/PhysRevB.47.2244 10006264

[B4] Zhu JL Yu JZ Li ZQ Kawasoe Y Exact solutions of two electrons in a quantum dot J Phys Condens Matter 1996 8 7857 10.1088/0953-8984/8/42/005

[B5] Mikhailov ID Betancur FJ Energy spectra of two particles in a parabolic quantum dot: numerical sweep method Phys stat sol (b) 1999 213 325 332 10.1002/(SICI)1521-3951(199906)213:2<325::AID-PSSB325>3.0.CO;2-W

[B6] Peeters FM Schweigert VA Two-electron quantum disks Phys Rev B 1996 53 1468 1474 10.1103/PhysRevB.53.1468 9983608

[B7] Mikhailov ID Marín JH García F Off-axis donors in quasi-two-dimensional quantum dots with cylindrical symmetry Phys stat sol (b) 2005 242 8 1636 1649 10.1002/pssb.200540053

[B8] Betancur FJ Mikhailov ID Oliveira LE Shallow donor states in GaAs-(Ga, Al)As quantum dots with different potential shapes J Appl Phys D 1998 31 3391 10.1088/0022-3727/31/23/013

